# The Embryonic *mir-35* Family of microRNAs Promotes Multiple Aspects of Fecundity in *Caenorhabditis elegans*

**DOI:** 10.1534/g3.114.011973

**Published:** 2014-07-21

**Authors:** Katherine McJunkin, Victor Ambros

**Affiliations:** Program in Molecular Medicine, RNA Therapeutics Institute, University of Massachusetts Medical School, Worcester, Massachusetts 01606

**Keywords:** fertility, maternal effect, germline, sperm, male fertility

## Abstract

MicroRNAs guide many aspects of development in all metazoan species. Frequently, microRNAs are expressed during a specific developmental stage to perform a temporally defined function. The *C. elegans mir-35-42* microRNAs are expressed abundantly in oocytes and early embryos and are essential for embryonic development. Here, we show that these embryonic microRNAs surprisingly also function to control the number of progeny produced by adult hermaphrodites. Using a temperature-sensitive *mir-35-42* family mutant (a deletion of the *mir-35-41* cluster), we demonstrate three distinct defects in hermaphrodite fecundity. At permissive temperatures, a mild sperm defect partially reduces hermaphrodite fecundity. At restrictive temperatures, somatic gonad dysfunction combined with a severe sperm defect sharply reduces fecundity. Multiple lines of evidence, including a late embryonic temperature-sensitive period, support a role for *mir-35-41* early during development to promote subsequent sperm production in later larval stages. We further show that the predicted *mir-35* family target *sup-26 (suppressor-26)* acts downstream of *mir-35-41* in this process, suggesting that s*up-26* de-repression in *mir-35-41* deletion mutants may contribute to temperature-sensitive loss of fecundity. In addition, these microRNAs play a role in male fertility, promoting proper morphogenesis of male-specific mating structures. Overall, our results demonstrate that robust activity of the *mir-35-42* family microRNAs not only is essential for embryonic development across a range of temperatures but also enables the worm to subsequently develop full reproductive capacity.

microRNAs are a class of endogenous 22-23-nucleotide RNAs that repress expression of complementary target mRNAs to govern diverse developmental and physiological processes in essentially all complex eukaryotes. In most cases, mature microRNAs are generated from much longer transcripts through a series of nucleolytic cleavages and subsequently loaded into complexes with Argonaute proteins (Ketting 2011). Together with the effector protein GW182, the microRNA-loaded Argonaute forms the RNA-induced silencing complex (miRISC), which inhibits the translation and/or stability of complementary target mRNAs. The seed region (nucleotides 2–7) of a microRNA is the most important for determining target specificity (Bartel 2009). microRNAs that share the same seed sequence are classified as a “family” because they can potentially bind and redundantly regulate the same set of target mRNAs.

The *C. elegans mir-35* family of microRNAs is abundantly expressed in oocytes and early embryos, and is essential for embryonic development (Lau *et al.* 2001; Wu *et al.* 2010; Alvarez-Saavedra and Horvitz 2010). This microRNA family consists of eight members (*mir-35-42*) that reside in two loci (*mir-35-41* and *mir-42-44*) (Figure 1A). Deletion of all eight *mir-35-42* microRNA genes results in slowed embryonic development culminating in completely penetrant embryonic or early larval lethality (Alvarez-Saavedra and Horvitz 2010). Strains that carry a deletion that only affects seven out of eight family members, *mir-35-41*(*nDf50*) or *mir-35-41*(*gk262*), and hence express only *mir-42*, display a partially penetrant embryonic lethality (Alvarez-Saavedra and Horvitz 2010; Liu *et al.* 2011; Massirer *et al.* 2012). For populations of *mir-35-41*(*nDf50*) or *mir-35-41*(*gk262*) embryos, the penetrance of lethality depends on the temperature at which the animals are grown, with lower frequency of lethality at 15° or 20° and nearly complete lethality at 25° (Alvarez-Saavedra and Horvitz 2010; Massirer *et al.* 2012). Thus, the *mir-35-41* deletion genotype (with only *mir-42* intact) can be considered hypomorphic for *mir-35* family function at permissive temperatures (15° or 20°) and a more severe loss of *mir-35* family function at a restrictive temperature (25°).

**Figure 1:**
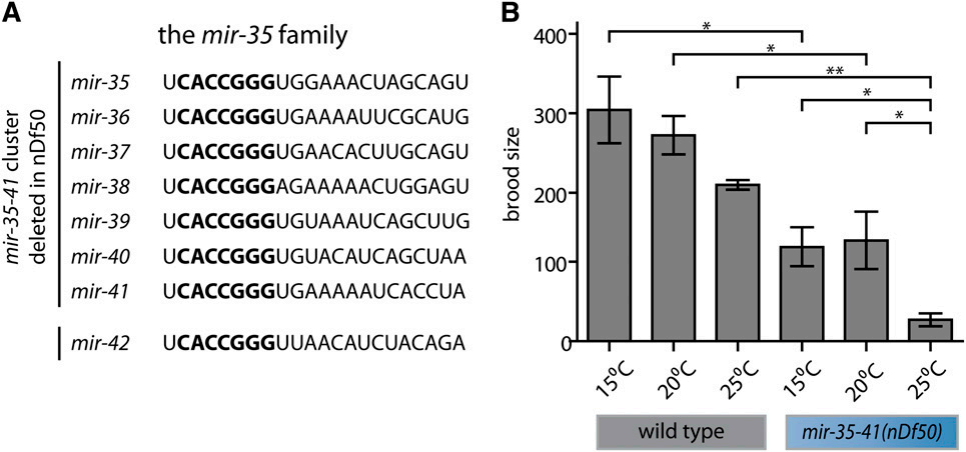
The *mir-35* family promotes hermaphrodite fecundity. (A) Mature microRNA sequences of all members of the *mir-35-42* seed family. Seed sequence is in bold. (B) Brood size (y-axis) represents the number of progeny produced per hermaphrodite, including dead embryos and larvae, as well as living progeny. Wild-type or *mir-35-41(nDf50)* animals were grown at the temperature indicated. Mean and SE are plotted. *p-value < 0.05, **p-value < 0.01, two-tailed Student’s *t*-test.

Because *mir-35-41* deletion mutants can bypass embryonic lethality at 15° or 20°, postembryonic phenotypes have also been characterized for animals that escape lethality at these permissive temperatures. These viable *mir-35-41* mutant animals display reduced proliferation of the intestine and the mitotic germline, which may result from de-repression of *lin-23* and *gld-1*, respectively (Liu *et al.* 2011). Interestingly, *mir-35-41* animals are also enhanced in their response to exogenous RNAi, an effect that depends on an indirect downregulation of *lin-35/Rb* (Massirer *et al.* 2012).

Here, we examined the effect of *mir-35-41* deletion on hermaphrodite fecundity. We show that the *mir-35* family acts in multiple processes, in both the germline and the soma, to promote full reproductive capacity. In particular, we provide evidence that these embryonically expressed microRNAs act early during development to promote spermatogenesis in subsequent larval stages. We also show that *suppressor-26* (*sup-26*), a predicted *mir-35* family target gene encoding an RNA-binding protein, acts downstream of *mir-35-41* in this context.

## Materials and Methods

### *C. elegans* culture and phenotypic characterization

*C. elegans* were cultured on NGM seeded with HB101. Strains were maintained at 15° or 20° for 72 hr or 25° for 48 hr prior to beginning experiments conducted at the respective temperatures. For quantification of brood size, single L4 hermaphrodites were placed on individual 3-cm NGM plates for approximately 24 hr. Animals were moved to a fresh plate each day until progeny were no longer produced. Approximately 24 hr after removal of the parent, larvae and embryos were counted on each plate. For brood size quantification when mating with wild-type males, five males and one L4 hermaphrodite were added to a 3-cm NGM plate and transferred to a fresh plate each day until progeny were no longer produced.

Mating efficiency of *mir-35-41(nDf50);him-8(e1489)* males was assessed by placing one male with one *fog-2(q71)* L4 female on a 3-cm NGM plate. Plates containing progeny after 5 d were counted as successful matings. Mating efficiency of *mir-35-41(nDf50);him-8(e1489)* was normalized to *him-8* mating efficiency. Sperm dissection and *in vitro* activation were performed as described (Singaravelu *et al.* 2011).

The *mIn1* balancer marked with *mIs14* (*myo2*::*GFP*, *pes-10*::*GFP*, *F2B7.9*::*GFP)* was used to balance *mir-35-41(nDf50)*. To generate *mir-35-41(m+z-)* animals, GFP-negative animals were segregated from *mir-35-41(nDf50)/mIn1* mothers. To generate *mir-35-41(m-z+)* animals, *mir-35-41(nDf50)* hermaphrodites were crossed to males containing *mIn1*, and GFP-positive progeny were isolated. The balancer *qC1* marked with *qIs26 (rol-6(su1006)*, *lag-2*::*GFP)* was used to balance *sup-26(lf)* alleles in the *mir-35-41(nDf50)* background. To generate *mir-35-41(nDf50);sup-26(m^+^z^lf^)* animals, non-Rol progeny that segregated from *mir-35-41(nDf50);sup-26(lf)/qC1* mothers were isolated. To generate *mir-35-41(nDf50);sup-26(m^lf^z^+^*) animals, *mir-35-41(nDf50);sup-26(lf)* hermaphrodites were crossed to *mir-35-41(nDf50)* males; after the appearance of male cross progeny, hermaphrodite larval progeny were picked.

For determining the temperature-sensitive period of *mir-35-41(nDf50)* fecundity, a mixed-stage population of *mir-35-41(nDf50)* animals containing a *lag-2*::*GFP* reporter (*qIs56*) marking the distal tip cells was shifted to 25° for 12 hr. At the end of that period, individual animals were isolated and their developmental stages were determined by scoring the positions of the GFP-marked distal tip cells; accordingly, for each animal the approximate period of larval development spent at 25° was inferred from previously described rates of *C. elegans* gonadal development (Byerly *et al.* 1976). The brood of each animal was subsequently quantified during the adult stage.

For quantifying endomitotic oocytes, animals were maintained at 25° for least 48 hr before picking late L4 larvae or young (pre-gravid) adults. DAPI staining was performed the next day. Whole animals were fixed in 95% ethanol containing 500 ng/ml DAPI for 3 min at room temperature. For counting total spermatids, adults were harvested either at a pre-gravid stage or at an early gravid stage (14 hr after selection as late L4 larvae at 25°). After DAPI staining, Z-stacks (0.4-µm sections) were acquired of whole spermathecae. Spermatids were counted manually on 3D reconstructions of the spermathecae using the 4D viewer in MetaMorph Image Analysis Software (Sunnyvale, California).

### qPCR and 3′ RACE

For *sup-26* mRNA qPCR, embryo RNA samples were prepared by growing strains on egg media on NGM plates. Strains were shifted to 25° for 24 hr prior to isolating embryos by bleaching. 3′ RACE was performed from staged RNA samples using primers designed according to the SMART protocol from Clontech.

### Generation of transgenics

A 1.5-kb fragment of sequence upstream of the *sup-26* coding sequence was amplified using the primers CCTGGGTAGCTATTTCGTACGTAGTC and AAGATGCGTTCATTCTTGAATTATTATG tagged with an attB4 or attB1r site, respectively. The *sup-26* 3′ UTR was amplified with the following primers: ATGGACAGGACAACGTCTTCACTCCAC and AAAACTGCAAGACCAATCAGCGATTC tagged with attB2r or attB3 sites, respectively. The PCR products were cloned into pCFJ210 using MultiSite Gateway cloning (Life Technologies, Green Island, NY). Quickchange mutagenesis was performed on the entry clone containing the *sup-26* 3′ UTR using the primers CATCCACCGTTCCGTCATCGTCG and CTGCCGAGGAAAGGAGAATGAGTG to mutate the putative *mir-35* family binding site. Single-copy transgenes were generated as described (Frøkjaer-Jensen *et al.* 2008).

## Results

### *mir-35-41* promotes spermatogenesis

We noticed that *mir-35-41(nDf50)* hermaphrodites lay many unfertilized oocytes, a phenotype that can be symptomatic of reduced fecundity because of a sperm defect (Argon and Ward 1980). To measure fecundity of the *mir-35-41(nDf50)* strain, we counted the number of progeny produced by each hermaphrodite throughout its lifetime, including both live progeny and dead embryos and larvae. At permissive temperatures (15° or 20°), *mir-35-41(nDf50)* hermaphrodites produce fewer progeny than wild-type animals (Figure 1B). When animals were raised at 25°, *mir-35-41(nDf50)* hermaphrodites produced dramatically fewer progeny than at 20° or wild-type animals raised at 25° (Figure 1B). Therefore, *mir-35-41(nDf50)* has a moderate effect on hermaphrodite fecundity at a permissive temperature (20°) and a severe effect at a restrictive temperature (25°).

**Figure 2:**
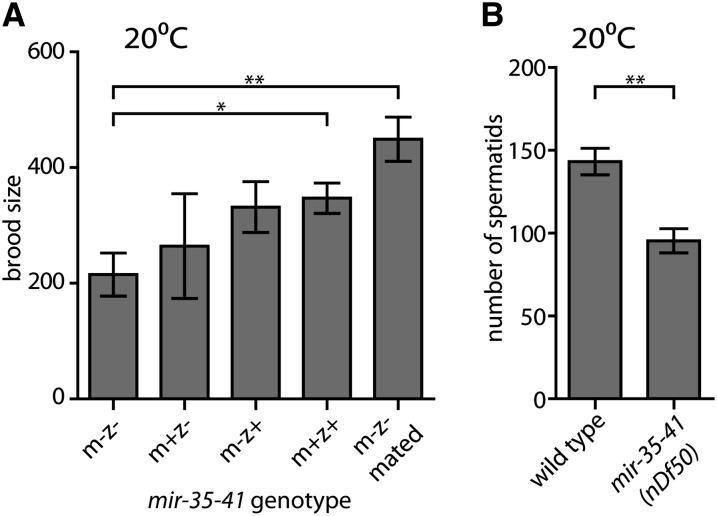
*mir-35-41(nDf50)* hermaphrodite fecundity at permissive temperature is rescued by zygotic *mir-35-41* expression or mating to wild-type males. (A) Brood size (y-axis) represents the number of progeny produced per hermaphrodite, including dead and living progeny. m = maternal *mir-35-41(nDf50)* genotype; z = zygotic genotype. mated = single hermaphrodite was mated to five wild-type males. (B) Number of spermatids per spermatheca in pre-gravid adult hermaphrodites of indicated genotype maintained at 20°. (A and B) Mean and SE are plotted. *p-value < 0.05, **p-value < 0.01, two-tailed Student’s *t*-test.

First, we further characterized the nature of the fecundity phenotype at a permissive temperature. Previous work demonstrated that *mir-35* family function in embryonic viability can be rescued by either maternal or zygotic *mir-35* family expression (Alvarez-Saavedra and Horvitz 2010). Similarly, we observed that fecundity of *mir-35-41(nDf50)* mutant hermaphrodites was partially rescued by maternal *mir-35-41* expression and fully rescued by zygotic expression at 20° (Figure 2A).

In wild-type *C. elegans* hermaphrodites, the number of sperm produced during larval development limits self-progeny to ∼300. However, wild-type hermaphrodites can generate up to ∼1000 progeny when mated to males, which provide additional sperm. To determine whether insufficient functional sperm could be responsible for reduced fecundity of *mir-35-41(nDf50)* hermaphrodites at a permissive temperature, *mir-35-41(nDf50)* hermaphrodites were crossed with wild-type males. At 20°, *mir-35-41(nDf50)* brood size was rescued by mating, indicating that a sperm defect underlies the brood size phenotype at this temperature (Figure 2A, last bar). We examined the number of sperm produced by *mir-35-41(nDf50)* hermaphrodites at 20° and found that *mir-35-41(nDf50)* hermaphrodites generate fewer spermatids than wild-type (Figure 2B). Thus, *mir-35-41* promotes maximal hermaphrodite spermatogenesis at 20°.

### *mir-35-41* promotes male fertility and tail morphogenesis

Because *mir-35-41* promotes spermatogenesis in hermaphrodites, we investigated whether *mir-35-41(nDf50)* males are fertile. To this end, a mutation that causes a high incidence of males (*him-8(e1489)*) through impaired X chromosome segregation was introduced to *mir-35-41(nDf50)*. Spermatids produced by *mir-35-41(nDf50);him-8(e1489)* males are numerous, appear normal, and can be activated *in vitro* (Supporting Information, Figure S1A). However, the mating efficiency of *mir-35-41(nDf50);him-8(e1489)* males is only 60% that of *him-8(e1489)*. We hypothesize that the low mating efficiency is largely due to defects in male-specific somatic structures. In 5% of *mir-35-41(nDf50);him-8(e1489)* males, tail structures essential for mating are highly abnormal (Figure S1B). Less apparent, milder tail defects may be present at higher penetrance and contribute to the large reduction in *mir-35-41(nDf50);him-8(e1489)* male mating efficiency. At 25°, *him-8(e1489)* alone exhibited temperature-sensitive phenotypes, so *mir-35-41(nDf50);him-8(e1489)* male phenotypes were only scored at 20°.

### Functions of the somatic gonad and spermatids are impaired in *mir-35-41(nDf50)* hermaphrodites at a restrictive temperature

**Figure 3:**
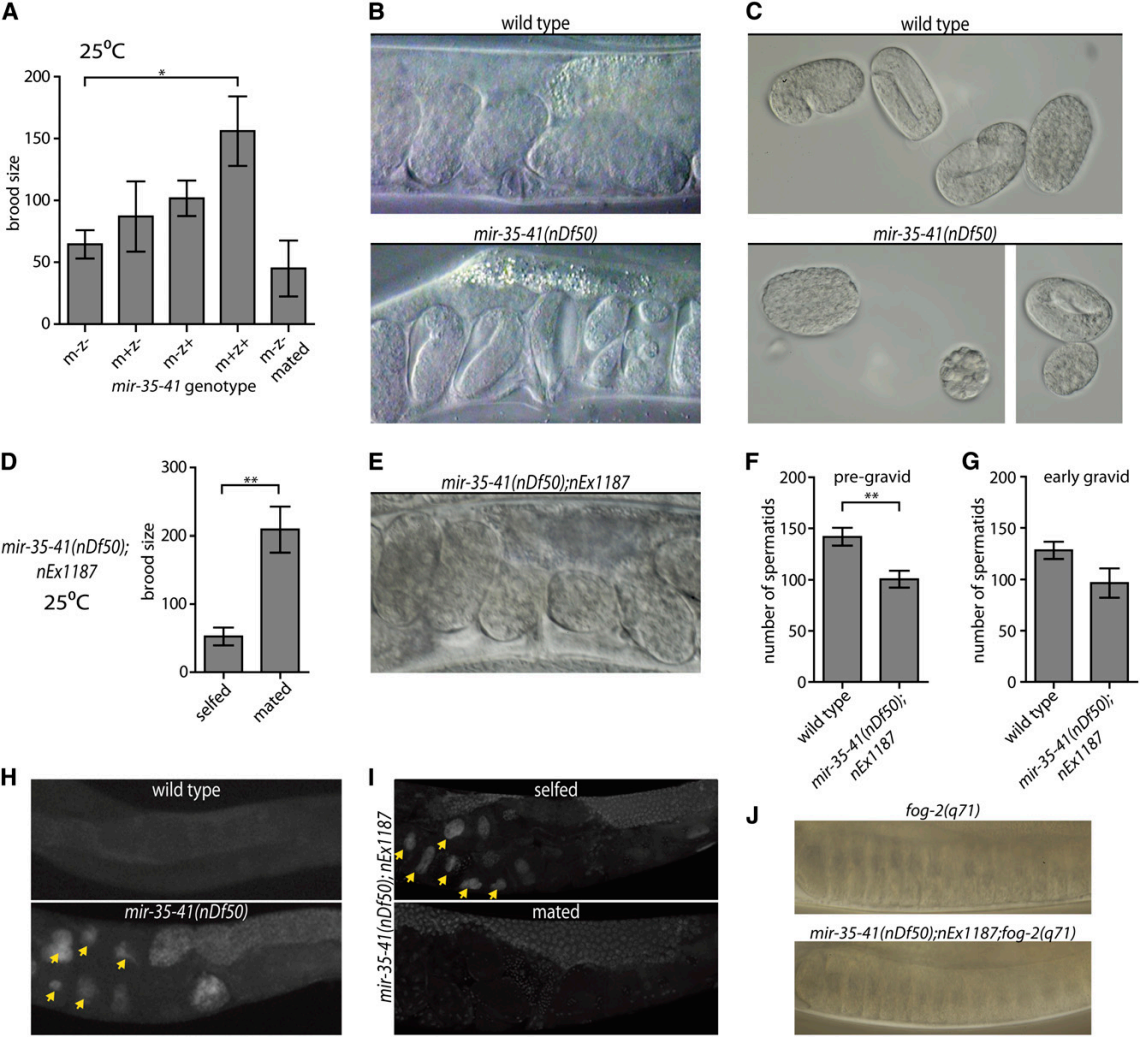
Both somatic gonad and sperm defects contribute to temperature-sensitive loss of fecundity in *mir-35-41(nDf50)* hermaphrodites. (A, D, F, G) Mean and SE are plotted. *p-value < 0.05, **p-value < 0.01, two-tailed Student’s *t*-test. (A) Brood size (y-axis) represents the number of progeny produced per hermaphrodite. m = maternal *mir-35-41(nDf50)* genotype; z = zygotic genotype. mated = single hermaphrodite was mated to five wild-type males. (B) Proximal uteri of wild-type or *mir-35-41(nDf50)* hermaphrodites grown at 25°. *mir-35-41(nDf50)* uteri contain very late-stage embryos, indicative of an egg-laying defective phenotype (Egl). (C) Embryos of wild-type or *mir-35-41(nDf50)* hermaphrodites grown at 25°. The size and shape of *mir-35-41(nDf50)* embryos are sometimes abnormal, indicating a defect of somatic gonad architecture. (D) Number of progeny produced by *mir-35-41(nDf50)* hermaphrodites containing a somatically expressed *mir-35* transgene *(nEx1187)* when selfed or mated to wild-type males. (E) Uteri of *mir-35-41(nDf50);nEx1187*, indicating that somatic expression of *mir-35* rescues the Egl phenotype of *mir-35-41(nDf50)*. (F and G) Number of spermatids per spermatheca in adult hermaphrodites maintained at 25°. Adults were harvested at a (F) pre-gravid or (G) early gravid stage. (H and I) Whole mount DAPI staining in wild-type and *mir-35-41(nDf50)* hermaphrodites (H) or *mir-35-41(nDf50)* hermaphrodites containing the *mir-35* transgene (*nEx1187*) when selfed or mated to wild-type males (I). Arrowheads indicate endomitotic oocytes. (J) Proximal gonad of virgin *fog-2(q71)* females that are otherwise wild-type (top) or containing *mir-35-41(nDf50);nEx1187*. In both genotypes, oocyte nuclei are pushed close together as oocytes accumulate and stack in the germline.

Next, we examined the nature of the severe fecundity defect of *mir-35-41(nDf50)* hermaphrodites at a restrictive temperature (25°). First, we determined whether maternal or zygotic loss of *mir-35-41* was responsible for the temperature-sensitive fecundity phenotype. We observed that either maternal or zygotic *mir-35-41* expression could partially rescue fecundity, whereas both maternal and zygotic *mir-35-41* expression were required for complete rescue (Figure 3A). Notably, the maternal effect (as evidenced by partial rescue of the phenotype by maternal *mir-35-41* and incomplete rescue by zygotic *mir-35-41*) supports a model wherein *mir-35-41* acts early in the development of the hermaphrodite embryo to ensure the animal’s maximal fecundity.

To assess whether a sperm defect is responsible for the reduced fecundity of *mir-35-41(nDf50)* hermaphrodites at 25°, mutant hermaphrodites were grown at 25° and mated to wild-type males. In contrast to the fecundity phenotype at a permissive temperature, which was rescued by mating (see above), mating did not rescue the brood size of hermaphrodites grown at 25° (Figure 3A, last bar). Therefore, at 25°, additional sperm-independent defects, not present at 20°, contribute to the reduced fecundity of *mir-35-41(nDf50)* hermaphrodites.

We hypothesized that the inability to rescue fecundity at 25° by mating *mir-35-41(nDf50)* hermaphrodites could be due to dysfunction of the somatic gonad. Consistent with such somatic gonad dysfunction, *mir-35-41(nDf50)* hermaphrodites grown at 25° display an egg-laying defective phenotype and occasionally produce misshapen eggs (Figure 3, B and C) (Greenstein *et al.* 1994; Kovacevic and Cram 2010). Expression of *mir-35* from a transgenic extrachromosomal array (*nEx1187*) (Alvarez-Saavedra and Horvitz 2010) rescued both the Egl phenotype and the ability to produce large numbers of cross-progeny when mated (Figure 3, D and E). However, *nEx1187* did not rescue *mir-35-41(nDf50)* hermaphrodite self-fecundity at 25° (Figure 3F). Fecundity of *mir-35-41(nDf50)*; *nEx1187* hermaphrodites could only be restored by mating to wild-type males. Therefore, *mir-35-41(nDf50)* hermaphrodites at 25° display a somatic gonad defect (which is rescued by *nEx1187*) and also a severe sperm defect (which is not rescued by *nEx1187*). Our interpretation of these results is that the sperm defect is due to germline loss of *mir-35*, and thus cannot be rescued by *nEx1187* because expression from high-copy extrachromosomal arrays is silenced in the germline (Kelly *et al.* 1997); however, other interpretations for the failure of *nEx1187* to rescue the sperm defect are also possible.

To further characterize the sperm defect, we examined the number of spermatids produced by *mir-35-41(nDf50)* hermaphrodites at 25°. Although we observed a reduced number of spermatids in *mir-35-41(nDf50)* hermaphrodites compared with wild-type, the quantity was similar to that observed at 20°, and thus cannot account for the more severe loss of fecundity at 25° (Figure 3F). Therefore, we examined whether sperm activation might be affected in *mir-35-41(nDf50)* hermaphrodites at 25°.

A hallmark of all sperm activation mutants is the rapid loss of spermatids from the spermatheca. Hermaphrodite spermatids are stored in the spermatheca and activated when they are pushed into the uterus by ovulation of the first oocyte (L’Hernault 2006). Activation results in the formation of a pseudopod, which allows mature spermatozoa that fail to fertilize the oocyte to “swim upstream” back to the spermatheca after each round of ovulation. Un-activated spermatids cannot return to the spermatheca and are thus pushed outside the body as embryos are laid. When spermatids were quantified from wild-type or *mir-35-41(nDf50);nEx1187* hermaphrodites after the first few rounds of ovulation, a dramatic loss of spermatids from *mir-35-41(nDf50);nEx1187* spermathecae was not observed (Figure 3G). Thus, *mir-35-41(nDf50);nEx1187* sperm do not fail to activate at 25°. Therefore, the severely reduced fecundity at 25° may be due to inefficient fertilization by *mir-35-41(nDf50);nEx1187* spermatozoa.

Another characteristic of sperm-defective mutants is the appearance of oocytes with abnormally high DNA content and distended nuclei (Ward and Miwa 1978). These endomitotic oocytes result when oocytes exit diakinesis and replicate their DNA in the absence of fertilization or cytokinesis (Greenstein *et al.* 1994; Iwasaki *et al.* 1996). Consistent with the sperm-defective phenotype, we observed endomitotic oocytes in the uteri of both *mir-35-41(nDf50)* (47.8% of uteri on the first day of gravidity, n = 113) and *mir-35-41(nDf50);nEx1187* (57.1%, n = 42) hermaphrodites grown at 25° (Figure 3, H and I). Importantly, endomitotic oocytes were completely absent from the germlines of 25° *mir-35-41(nDf50);nEx1187* hermaphrodites mated to wild-type males (0%, n = 40) (Figure 3I). Thus, endomitotic oocytes are a phenotypic trait of the sperm defect in *mir-35-41(nDf50)* and *mir-35-41(nDf50);nEx1187* hermaphrodites at 25°.

Although endomitotic oocytes arise due to relatively rare, stochastic ovulation in the absence of functional sperm (Ward and Miwa 1978; McCarter *et al.* 1997), the phenotype may be exacerbated if *mir-35-41* also contributes to the inhibition of oocyte meiotic maturation in the absence of sperm (Greenstein *et al.* 1994; Govindan *et al.* 2006, 2009). If aberrant oocyte maturation contributes to the endomitotic oocyte phenotype observed in *mir-35-41(nDf50);nEx1187* at 25°, then oocytes in *mir-35-41(nDf50);nEx1187* females (which lack sperm) would aberrantly mature and fail to accumulate or “stack” in the proximal gonad. To generate females, we introduced a *feminization-of-germline* mutation (*fog-2(q71)*) into the *mir-35-41(nDf50);nEx1187* background. In *mir-35-41(nDf50);nEx1187;fog-2(q71)* females, oocytes stack similarly to *fog-2(q71)* females (Figure 3J). Therefore, the inhibition of oocyte meiotic maturation is normal in *mir-35-41(nDf50);nEx1187;fog-2(q71)*, and a defect in this inhibition is thus unlikely to contribute to the endomitotic oocyte phenotype observed in *mir-35-41(nDf50);nEx1187* at 25°.

### *mir-35-41* acts early during hermaphrodite development to promote sperm function

**Figure 4:**
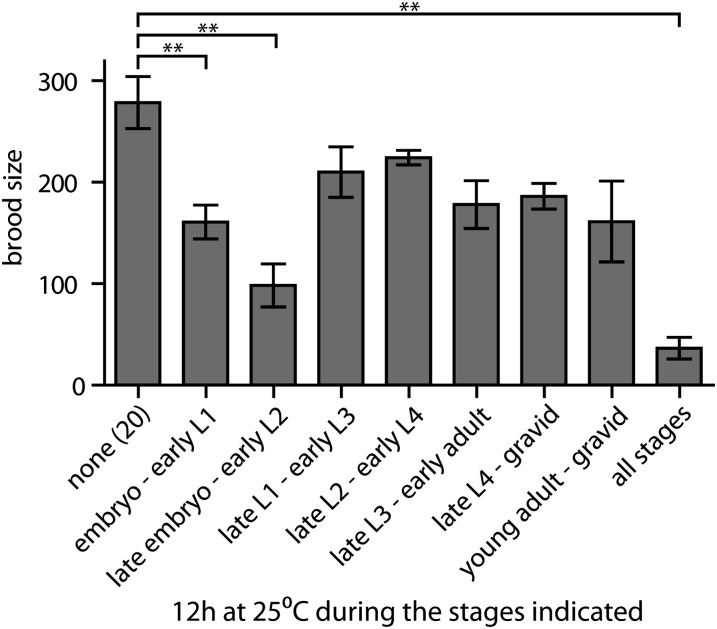
The temperature-sensitive period of *mir-35-41(nDf50)* hermaphrodite fecundity is prior to the L2 stage. Number of progeny from *mir-35-41(nDf50)* hermaphrodites shifted to 25° for 12 hr at the indicated larval stages. Mean and SE are plotted. **p-value < 0.01, two-tailed Student’s *t*-test.

We observed that the self-fecundity of *mir-35-41(nDf50)* hermaphrodites at restrictive temperature depends on contributions from both their mother’s genotype and from their own zygotic genotype (Figure 3A). This suggests that that the spermatogenesis defect of *mir-35-41(nDf50)* hermaphrodites could be caused (at least in part) by reduction of *mir-35* family function early in development. To further test this model, we determined the temperature-sensitive period of *mir-35-41(nDf50)* self-fecundity. Animals were shifted to 25° for a period of 12 hr at various stages of development. All animals that spent late embryogenesis at 25° displayed severely reduced fecundity (Figure 4). A much milder impact on fecundity was observed in animals shifted to 25° at any stage after L1. Therefore, *mir-35-41* acts early in development—perhaps during embryogenesis—to ensure the maximal fecundity of the animal when it matures to the adult. This is consistent with the temporal expression pattern of the *mir-35* family, primarily in oocytes and early embryos.

### SUP-26 contributes to the temperature-sensitive sperm defect in *mir-35-41(nDf50)*

Next, we looked for predicted *mir-35* family target genes that might play a role in hermaphrodite fecundity downstream of *mir-35-41*. Interestingly, the top prediction on Targetscan, *sup-26* (also known as *tag-310*), was previously implicated in sex-specific development (Jan *et al.* 2011). SUP-26 is an RNA-binding protein that promotes masculine sex determination by inhibiting translation of *tra-2* (*transformer-2*) mRNA in somatic tissues (Manser *et al.* 2002; Mapes *et al.* 2010). In the germline of developing hermaphrodite larvae, *tra-2* translational status controls when sperm or oocytes are generated. Stage-specific inhibition of germline *tra-2* translation by the RNA binding proteins FOG-2 and GLD-1 (defective in GermLine Development-1) drives spermatogenesis during the third and fourth larval stages (Ellis and Schedl 2007). The role of SUP-26 in the germline has not been examined. However, the previously described somatic role of SUP-26 in controlling *tra-2* translation makes *sup-26* an interesting candidate *mir-35* family target gene in the context of hermaphrodite fecundity. Multiple 3′ UTRs have been annotated for the *sup-26* mRNA (Mangone *et al.* 2010; Jan *et al.* 2011), only one of which contains the *mir-35* family target site (Figure S2A). For *sup-26* to be a direct *mir-35* family target, the longest 1146-bp 3′ UTR must be used. We performed 3′ Rapid Amplification of cDNA Ends (RACE) to determine which *sup-26* 3′ UTRs are present throughout development. The 1146-bp 3′ UTR was amplified from all samples and confirmed by sequencing, whereas products corresponding to the other annotated 3′ UTRs were not observed (Figure S2B). In addition, *sup-26* mRNA was significantly enriched in pull-down of the RISC effector proteins ALG-1-Interacting-1 (AIN-1) and AIN-2 (Zhang *et al.* 2007; Hammell *et al.* 2008). Thus, endogenous *sup-26* mRNA contains a highly conserved *mir-35* family target site and associates with miRISC.

**Figure 5:**
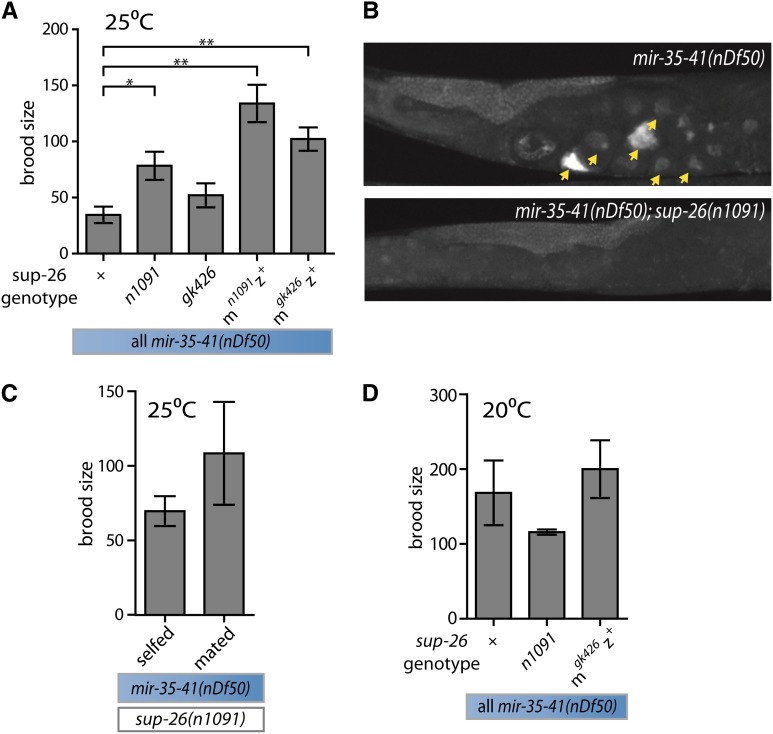
*sup-26* contributes to the temperature-sensitive sperm defect of *mir-35-41(nDf50)*, but not other aspects of *mir-35-41(nDf50)* reduced fecundity. (A) Number of progeny produced per hermaphrodite (grown at 25°). m = maternal; z = zygotic *sup-26* genotype. (B) DAPI staining. Arrowheads indicate endomitotic oocytes. (C) Number of self- or cross-progeny produced by *mir-35-41(nDf50);sup-26(n1091)* hermaphrodite at 25°. Wild-type sperm do not significantly increase fecundity. (D) Number of self-progeny produced per hermaphrodite (grown at 20°). *Sup-26(lf)* does not robustly affect *mir-35-41(nDf50)* fecundity at 20°. (A, C, D) Mean and SE are plotted. *p-value < 0.05, **p-value < 0.01, two-tailed Student’s *t*-test with Welch’s correction for unequal variance.

If *sup-26* is a *mir-35* family target gene whose de-repression in *mir-35-41(nDf50)* contributes to loss of fecundity, then *sup-26(lf)* might suppress one or more aspects of the *mir-35-41(nDf50)* fecundity phenotype. *mir-35-41(nDf50)*;*sup-26(lf)* animals exhibit incompletely penetrant embryonic lethality, so we assessed the fecundity of escaper *mir-35-41(nDf50)*;*sup-26(lf)* hermaphrodites that survived to adulthood. Strikingly, a weak (*n1091*) or strong (*gk426*) allele of *sup-26* partially suppressed the temperature-sensitive fecundity of *mir-35-41(nDf50)* at 25°, indicated by an increased number of hermaphrodite self-progeny in *mir-35-41(nDf50)*;*sup-26(lf)* (Figure 5A).

Because the number of self-progeny was increased in *mir-35-41(nDf50);sup-26(lf)* compared with *mir-35-41(nDf50)*, we hypothesized that the temperature-sensitive sperm defect was rescued by *sup-26(lf)*. Consistent with this, *sup-26(lf)* also suppressed the appearance of endomitotic oocytes in *mir-35-41(nDf50);sup-26(lf)* (Figure 5B). Only 6.8% of uteri in *mir-35-41(nDf50);sup-26(n1091)* contained endomitotic oocytes on the first day of gravidity (n = 74), compared with 47.8% in *mir-35-41(nDf50)*. Therefore, the predicted *mir-35* family target gene *sup-26* functions downstream of *mir-35-41* in regulating spermatogenesis at restrictive temperature.

Because the *mir-35-41(nDf50)* sperm phenotype at restrictive temperature is attributable, at least in part, to maternal loss of *mir-35-41*, we postulated that *mir-35-41* might act in the oocyte and early embryo to repress the maternal load of *sup-26* mRNA. If this is the case, then loss of maternal *sup-26* function would be predicted to suppress the *mir-35-41(nDf50)* self-fecundity defect at restrictive temperature. Removing the maternal contribution of wild-type *sup-26* mRNA (*sup-26(m^lf^z^+^)*) rescues the brood size of *mir-35-41(nDf50)* animals at 25°, at least as well as (and, in fact, better than) complete *sup-26* loss-of-function (*sup-26(m^lf^z^lf^)*) (Figure 5A). The rescue of *mir-35-41(nDf50)* by *sup-26(m^lf^z^+^)* to nearly wild-type fecundity suggests that the primary effect of *mir-35-41* on hermaphrodite fecundity is upstream of the maternal contribution of *sup-26*. This suggests that *mir-35-41* promotes sperm function by acting in the maternal germline and/or early embryo to limit the expression of SUP-26 from maternally supplied *sup-26* mRNA. Interestingly, *sup-26(m^lf^z^+^)* appeared to suppress *mir-35-41(nDf50)* brood size at 25° better than *sup-26(m^lf^z^lf^)* (Figure 5A), suggesting a second function for *sup-26* (in this case zygotically expressed) in promoting fecundity of *mir-35-41(nDf50)* embryos.

Because examining the translational status of maternally supplied *sup-26* mRNA (without detecting the zygotic contribution of the same transcript) is technically difficult, we examined total embryonic *sup-26* mRNA for evidence of *mir-35-41*–dependent regulation. Neither qPCR of endogenous *sup-26* nor GFP reporter transgenes showed evidence of *mir-35-41*–dependent or *sup-26* 3′ UTR–dependent control of mRNA abundance or translation in whole embryos (Figure S2, C and D). Thus, although we were unable to examine the maternal *sup-26* transcript alone, the bulk of zygotically transcribed *sup-26* mRNA does not appear to be subject to *mir-35-41* regulation (see *Discussion*).

In addition, we examined whether *sup-26* plays a role in the other aspects of *mir-35-41(nDf50)* loss of fecundity. In contrast to the sperm defect at a restrictive temperature, function of the somatic gonad is not rescued in *mir*-3*5-41(nDf50);sup-26(lf)* hermaphrodites, because they do not reproducibly produce large numbers of cross progeny when mated to wild-type males (Figure 5C). Furthermore, loss of *sup-26* function does not suppress the *mir-35-41(nDf50)* fecundity phenotype at 20° (Figure 5D). Therefore, only the defect in sperm function in *mir-35-41(nDf50)* at a restrictive temperature, but not the defect in spermatogenesis observed at a permissive temperature, depends on *sup-26*.

## Discussion

We have demonstrated that the *mir-35* family acts at multiple levels to promote hermaphrodite fecundity. By examining the phenotype of *mir-35-41(nDf50)* at multiple temperatures and the effects of a somatic *mir-35* rescue, we have delineated at least four ways in which *mir-35-41* promotes fecundity. In *mir-35-41(nDf50);him-8(e1489)* males, mating is impaired by abnormal development of the male-specific copulatory apparatus. In *mir-35-41(nDf50)* hermaphrodites, a moderate defect in spermatogenesis reduces the number of progeny produced at a permissive temperature. At a restrictive temperature, dysfunction of the somatic gonad and a severe defect in sperm function further reduce *mir-35-41(nDf50)* hermaphrodite fecundity. We propose that while the number of spermatids produced at both temperatures is reduced, inefficient fertilization further hampers the function of spermatids produced at restrictive temperature. Interestingly, *sup-26(lf)* only suppresses the low fecundity observed at restrictive temperature, indicating that *sup-26* may play a role in sperm function but not in sperm production.

The fact that adult fecundity is affected by loss of *mir-35-41* is intriguing in light of the expression pattern of the *mir-35* family: primarily in oocytes and early embryos. Here, we show that loss of *mir-35-41* causes early developmental defects that result in the observed adult phenotypes. In particular, our studies on the temperature-sensitive sperm defect support this model. First, zygotic *mir-35* expression does not fully rescue this phenotype, indicating a maternal effect of *mir-35-41* on fecundity of the adult. Second, the temperature-sensitive period of this phenotype is early in development (approximately late embryogenesis). Third, loss of the maternal contribution of *sup-26* rescues the fecundity of *mir-35-41(nDf50)* adults at a restrictive temperature. Together, these results strongly support the model that the molecular basis of this phenotype arises in early development.

The suppression of *mir-35-41(nDf50)* loss of self-fecundity at 25° by loss of maternal *sup-26* strongly suggests that *mir-35-41* ensures fecundity by repressing maternal *sup-26* in the oocyte or early embryo. When examining zygotic *sup-26* endogenous transcripts and reporter transgenes, we did not see clear evidence of *mir-35-41*–dependent regulation. One possibility is that the maternal load of *sup-26* is at a sufficiently low concentration to be subject to *mir-35* family regulation, while strong zygotic transcription increases *sup-26* mRNA abundance beyond the threshold of repression. This is consistent with the expression pattern of *sup-26*, and also with that of *mir-35-42*, which is most abundant in oocytes and early embryos, decaying in levels throughout embryogenesis (Wu *et al.* 2010; Stoeckius *et al.* 2009). Alternatively, *mir-35-41* may act upstream of *sup-26* in an unknown indirect manner.

We do not yet understand how *sup-26* affects hermaphrodite fecundity downstream of *mir-35-41. Sup-26* is thought to modulate sex determination by inhibiting translation of *tra-2* mRNA in the soma (Manser *et al.* 2002; Mapes *et al.* 2010). Here, we show that *sup-26* can also affect germline development. If the germline effect of *sup-26* were also via translational inhibition of *tra-2*, then ectopic de-repression of maternal *sup-26* in *mir-35-41(nDf50)* would be expected to extend the period of *tra-2* inhibition, thus increasing rather than decreasing sperm production (Kuwabara *et al.* 1998). One possibility is that SUP-26 interacts with a different complement of proteins in the soma and the germline to cause *tra-2* activation in the germline and *tra-2* inhibition in the soma. It is also possible that SUP-26 binds and regulates additional unidentified RNA targets other than *tra-2*, and these interactions may account for the deleterious effect of SUP-26 on sperm function that we observe here. Identification of other target genes through an approach such as affinity purification of SUP-26 followed by deep sequencing of associated RNAs would be a key advance in our understanding of SUP-26 biology.

This work describes two new temperature-sensitive phenotypes of *mir-35-41(nDf50)*, in addition to the previously described temperature-sensitive embryonic lethality (Alvarez-Saavedra and Horvitz 2010). Although the basis of temperature sensitivity of these phenotypes is not fully understood, we hypothesize that microRNA target repression (at least by *mir-35* family microRNAs) is less efficient at 25° than at 20°. In this scenario, a single *mir-35-42* family member (*mir-42*) could be sufficient to repress the *mir-35-42* target genes at 20°, but insufficient at 25°. This model is consistent with the observation that the embryonic lethality phenotype of the *mir-35-42* deletion strain is completely penetrant at all temperatures (Alvarez-Saavedra and Horvitz 2010). Thus, this model provides a sound framework for conceptualization of the temperature-sensitive phenotypes observed in the *mir-35-41* deletion strain. The *mir-35* microRNA family may provide a fascinating setting in which to explore the effects of natural *in vivo* temperature changes on microRNA target recognition and/or repression.

The *mir-35* family is unique among microRNAs across diverse species for its strong maternal effect. In vertebrates, microRNAs that are essential for embryogenesis, such as miR-430 in zebrafish and miR-290-295 in mice, are expressed abundantly only after the onset of zygotic transcription (Giraldez *et al.* 2006; Medeiros *et al.* 2011). Thus, the *mir-35* family may represent a novel paradigm for microRNA control of embryogenesis. The maternal and early embryonic expression of *mir-35-42* suggests that its targets may be repressed at the earliest stages of embryogenesis and later released from repression. Our studies of *sup-26* are consistent with this model. Thus, future studies of *mir-35* family function may have broad implications for our understanding of the reversibility of microRNA target repression and the full complement of microRNA functions during development.

## Supplementary Material

Supporting Information
