# Supplementary material for: The Embryonic mir-35 Family of microRNAs Promotes Multiple Aspects of Fecundity in Caenorhabditis elegans
Source: G3 (Bethesda). 2014 Jul 21;4(9):1747–54. doi: 10.1534/g3.114.011973 (PMC4169167; doi:10.1534/g3.114.011973)
Supplement: Supporting Information [file supp_4_9_1747__index.html]

The Embryonic mir-35 Family of microRNAs Promotes Multiple Aspects of Fecundity in Caenorhabditis elegans — Supporting Information 

# The Embryonic *mir-35* Family of microRNAs Promotes Multiple Aspects of Fecundity in *Caenorhabditis elegans*

## Supporting Information for McJunkin and Ambros, 2014

**Files in this Data Supplement:**

- Supporting Information - Figures S1-S2 (PDF, 670 KB)
- Figure S1 - *mir-35-41(nDf50);him-8(e1489)* males produce normal sperm, but abnormal male tail structures. (PDF, 460 KB)
- Figure S2 - Endogenous *sup-26* mRNA contains a *mir-35* family target site, but *sup-26* Q-PCR and transcriptional reporters do not show *mir-35-41*-dependent regulation in embryos. (PDF, 596 KB)
