# Supplementary material for: The Embryonic mir-35 Family of microRNAs Promotes Multiple Aspects of Fecundity in Caenorhabditis elegans
Source: G3 (Bethesda). 2014 Jul 21;4(9):1747–54. doi: 10.1534/g3.114.011973 (PMC4169167; doi:10.1534/g3.114.011973)
Supplement: Supporting Information [file supp_g3.114.011973_011973SI.pdf]

G3 · Genes | Genomes | Genetics

The embryonic *mir-35-42* family of microRNAs promotes multiple aspects of hermaphrodite fecundity in *C. elegans*

Katherine McJunkin* and Victor Ambros*

*Program in Molecular Medicine, RNA Therapeutics Institute, University of Massachusetts Medical School, Worcester, MA 01606, USA

Corresponding authors:
Katherine McJunkin and Victor Ambros
373 Plantation Street
Biotech II Suite 306
Worcester, MA 01606
katherine.mcjunkin@umassmed.edu
victor.ambros@umassmed.edu

DOI: 10.1534/g3.114.011973

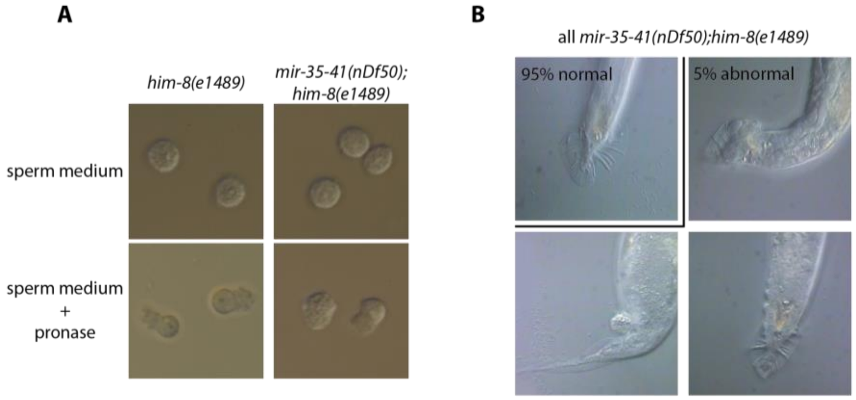

Figure S1 ***mir-35-41(nDf50)*;*him-8(e1489)* males produce normal sperm, but abnormal male tail structures.** (A) Sperm dissected from *him-8(e1489)* or *mir-35-41(nDf50)*;*him-8(e1489)* males. Sperm of either genotype were activated to form pseudopods by *in vitro* treatment with pronase (bottom). (B) Representative tail structures of *mir-35-41(nDf50)*;*him-8(e1489)* males. The majority of animals are wild type (upper left), while approximately five percent display grossly abnormal tails incompatible with mating.

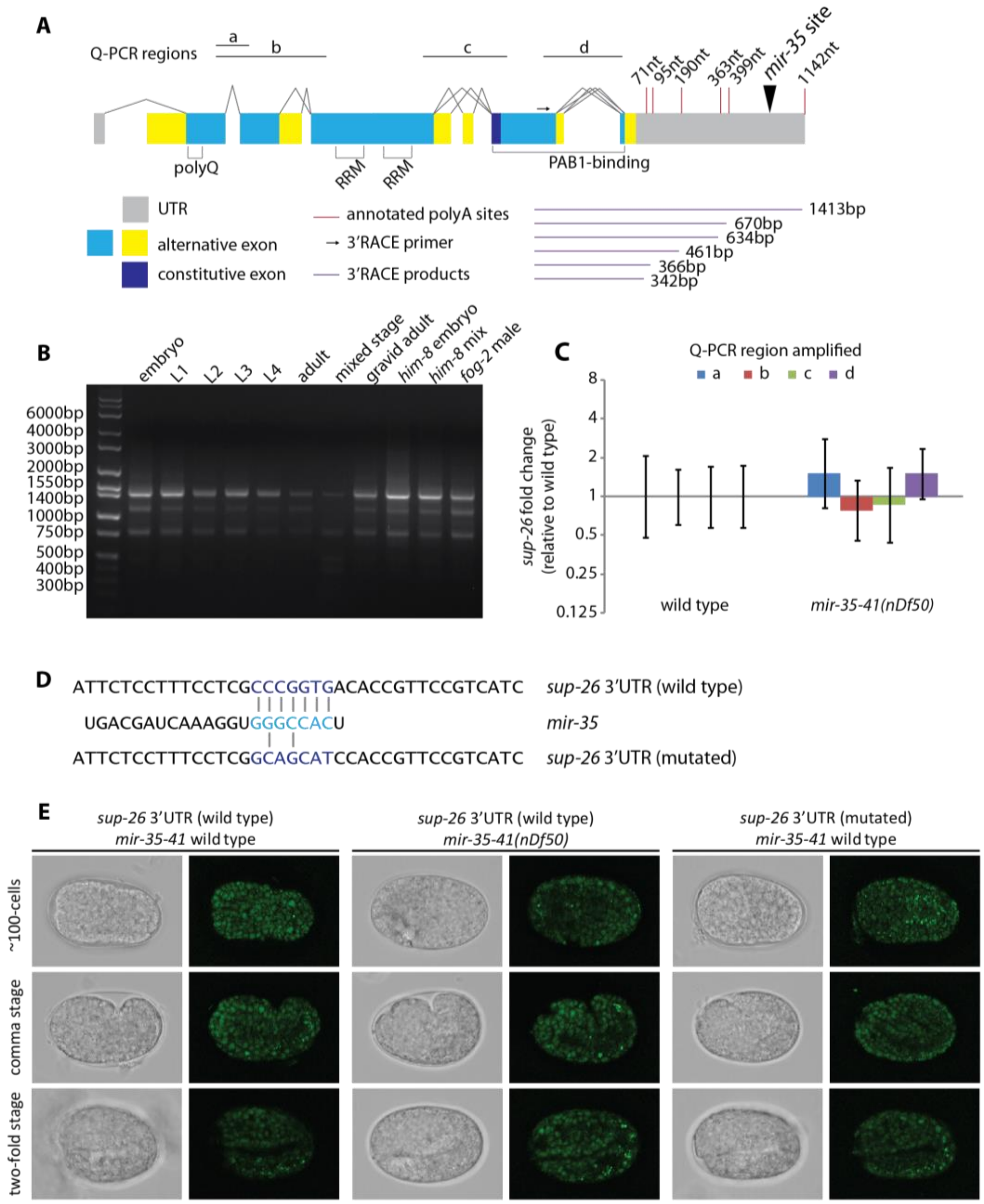

Figure S2 Endogenous *sup-26* mRNA contains a *mir-35* family target site, but *sup-26* Q-PCR and transcriptional reporters do not show *mir-35-41*-dependent regulation in embryos. (A) Schematic of annotated *sup-26* 3'UTRs and predicted 3'RACE products. (B) *sup-26* 3' RACE products. The prominent 1413bp product corresponds to the 1142nt 3'UTR containing the putative *mir-35* target site. (C) Q-PCR of *sup-26* mRNA in wild type or *mir-35-41(nDf50)* embryos maintained at 25°. Location of Q-PCR primers are indicated on gene model in (A). (D) *mir-35* family target site in wild type or mutated *sup-26* 3'UTR. (E) DIC and GFP fluorescence micrographs of transgenic embryos at indicated stages containing $sup\text{-}26^{promoter}$*::GFP-H2B::*$sup\text{-}26^{3'UTR}$ transcriptional fusion reporters. The 3'UTR corresponds to either the wild type *sup-26* 3'UTR or a mutated version as indicated in (D). Animals are either wild type or *mir-35-41(nDf50)* as indicated. Animals were maintained at 25°.
