# Supplementary material for: The Embryonic mir-35 Family of microRNAs Promotes Multiple Aspects of Fecundity in Caenorhabditis elegans
Source: G3 (Bethesda). 2014 Jul 21;4(9):1747–54. doi: 10.1534/g3.114.011973 (PMC4169167; doi:10.1534/g3.114.011973)
Supplement: Supporting Information [file supp_g3.114.011973_FigureS1.pdf]

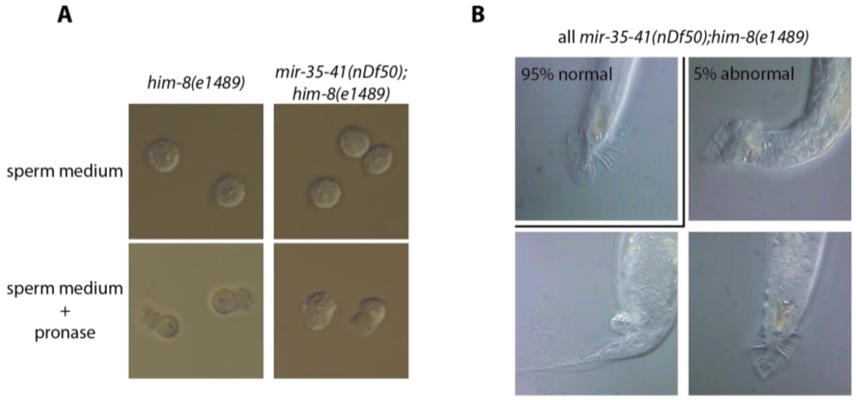

Figure S1 ***mir-35-41(nDf50)*;*him-8(e1489)* males produce normal sperm, but abnormal male tail structures.** (A) Sperm dissected from *him-8(e1489)* or *mir-35-41(nDf50)*;*him-8(e1489)* males. Sperm of either genotype were activated to form pseudopods by *in vitro* treatment with pronase (bottom). (B) Representative tail structures of *mir-35-41(nDf50)*;*him-8(e1489)* males. The majority of animals are wild type (upper left), while approximately five percent display grossly abnormal tails incompatible with mating.
