# Supplementary material for: The Embryonic mir-35 Family of microRNAs Promotes Multiple Aspects of Fecundity in Caenorhabditis elegans
Source: G3 (Bethesda). 2014 Jul 21;4(9):1747–54. doi: 10.1534/g3.114.011973 (PMC4169167; doi:10.1534/g3.114.011973)
Supplement: Supporting Information [file supp_g3.114.011973_FigureS2.pdf]

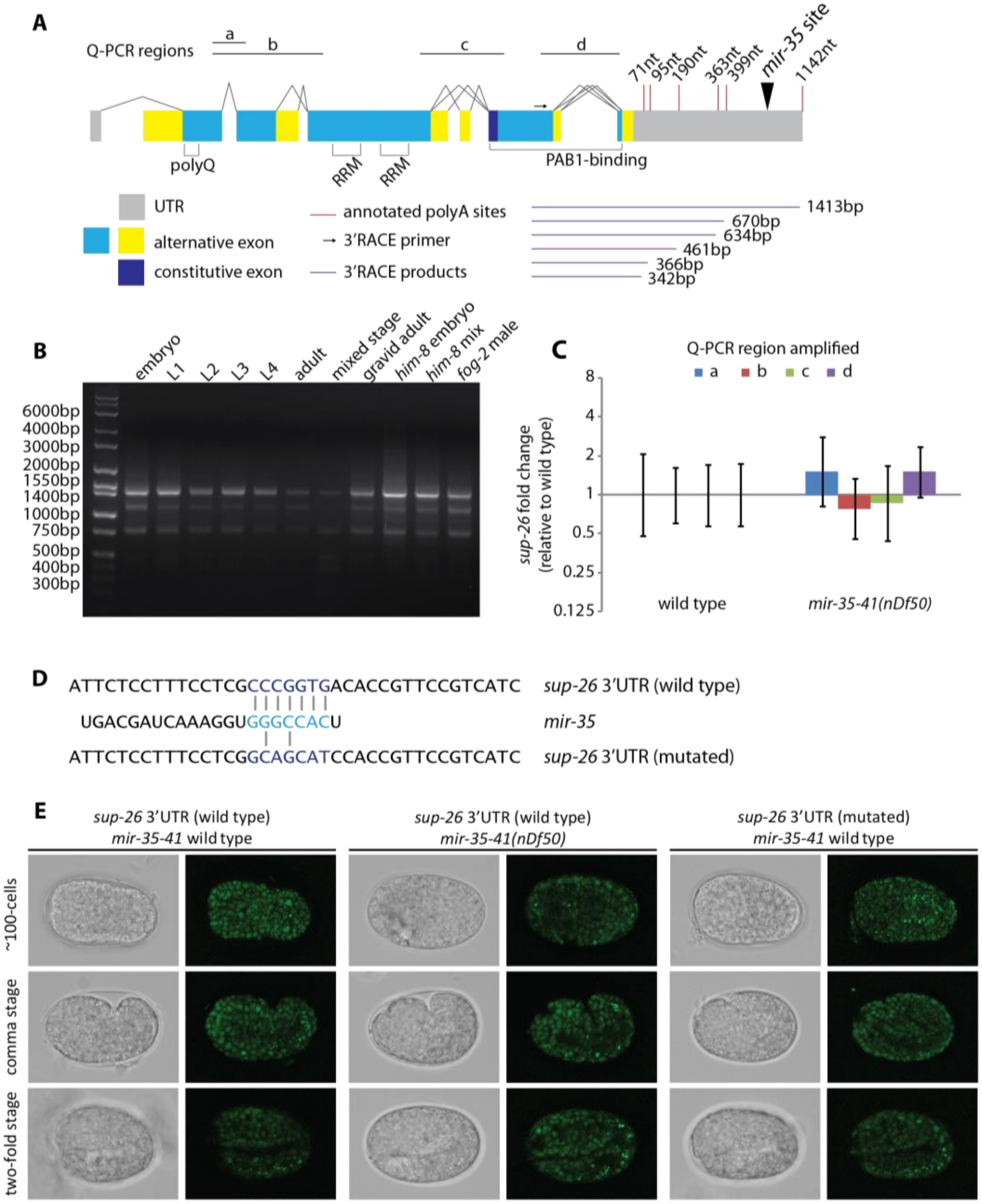

Figure S2 Endogenous *sup-26* mRNA contains a *mir-35* family target site, but *sup-26* Q-PCR and transcriptional reporters do not show *mir-35-41*-dependent regulation in embryos. (A) Schematic of annotated *sup-26* 3'UTRs and predicted 3'RACE products. (B) *sup-26* 3' RACE products. The prominent 1413bp product corresponds to the 1142nt 3'UTR containing the putative *mir-35* target site. (C) Q-PCR of *sup-26* mRNA in wild type or *mir-35-41(nDf50)* embryos maintained at 25°. Location of Q-PCR primers are indicated on gene model in (A). (D) *mir-35* family target site in wild type or mutated *sup-26* 3'UTR. (E) DIC and GFP fluorescence micrographs of transgenic embryos at indicated stages containing $\textit{sup-26}^{\textit{promoter}}$*::GFP-H2B::*$\textit{sup-26}^{\textit{3'UTR}}$ transcriptional fusion reporters. The 3'UTR corresponds to either the wild type *sup-26* 3'UTR or a mutated version as indicated in (D). Animals are either wild type or *mir-35-41(nDf50)* as indicated. Animals were maintained at 25°.
